# Catheter-Related Bloodstream Infection with *Leclercia adecarboxylata* and *Enterobacter cloacae* Complex Co-Infection: A Case Report and Literature Review

**DOI:** 10.3390/microorganisms14020402

**Published:** 2026-02-08

**Authors:** Po-Hsiu Huang, Po-Yu Liu, Hsien-Po Huang

**Affiliations:** 1 Division of Infectious Diseases, Department of Internal Medicine, Taichung Veterans General Hospital, Taichung 407, Taiwan; henry830104@gmail.com (P.-H.H.); liupoyu@gmail.com (P.-Y.L.); 2 School of Medicine, National Yang Ming Chiao Tung University, Taipei 112, Taiwan; 3 Department of Post-Baccalaureate Medicine, College of Medicine, National Chung Hsing University, Taichung 402, Taiwan

**Keywords:** *Leclercia adecarboxylata*, *Enterobacter cloacae* complex, catheter-related bloodstream infection, co-infection

## Abstract

Catheter-related bloodstream infections (CRBSIs) caused by *Leclercia adecarboxylata* are uncommon, and polymicrobial cases are even rarer. We report the first documented case caused by co-infection with *Leclercia adecarboxylata* and *Enterobacter cloacae* complex (ECC) in a woman with breast cancer undergoing chemotherapy through an indwelling chemoport. Antimicrobial susceptibility testing revealed that both isolates were susceptible to β-lactams, quinolones, and aminoglycosides. The patient achieved complete clinical recovery following intravenous ciprofloxacin therapy and prompt removal of the chemoport. This case highlights the emerging clinical relevance of *Leclercia adecarboxylata* and *Enterobacter cloacae* complex as potential pathogens capable of causing polymicrobial bloodstream infections in immunocompromised hosts and underscores the importance of considering rare environmental Gram-negative organisms as potential causes of catheter-related infections, particularly in patients with malignancy or long-term vascular access.

## 1. Introduction

Catheter-related bloodstream infections (CRBSIs) are frequent complications in patients with long-term vascular devices, usually caused by skin commensals such as *Staphylococcus epidermidis* and *Staphylococcus aureus* [1]. In contrast, infections due to environmental or opportunistic Gram-negative bacteria remain rare but clinically significant, especially in immunocompromised hosts.

*Leclercia adecarboxylata*, a member of the *Enterobacteriaceae* family, has recently emerged as an opportunistic pathogen associated with a variety of infections, including polymicrobial bloodstream infections and catheter-related bacteremia. Although infections due to *L. adecarboxylata* remain rare, they are increasingly reported in patients with malignancy, chronic illness, or indwelling vascular devices [2]. *Enterobacter cloacae* complex (ECC) is a Gram-negative pathogen and a common cause of healthcare-associated infections, including bloodstream infections, pneumonia, urinary tract infections, and device-related infections. ECC infections predominantly occur in hospitalized and immunocompromised patients and are frequently associated with indwelling intravascular catheters and prolonged healthcare exposure [3].

Despite the increasing recognition of *L. adecarboxylata* as an opportunistic pathogen and the well-known role of ECC as a pathogenic organism, co-infection involving these two organisms in catheter-related bloodstream infection has not been previously described. Here, we report the first documented case of chemoport-related bloodstream infection caused by co-infection with *L. adecarboxylata* and *Enterobacter cloacae* complex, highlighting its diagnostic and clinical implications.

## 2. Case Presentation

A 54-year-old woman with breast cancer was receiving regular chemotherapy via an implanted chemoport. She presented to the emergency department in September 2025 with a three-day history of fever and chills. She worked as a housekeeper and occasionally engaged in gardening activities. As her fever progressed, she was admitted for further evaluation. On admission, her vital signs were as follows: blood pressure 128/83 mmHg, temperature 38.4 °C, pulse rate 94 beats/min, and respiratory rate 20 breaths/min. Physical examination revealed no abnormalities of the head, eyes, ears, nose, or throat. There was no evidence of clubbing, cyanosis, lymphadenopathy, or jaundice. Pulmonary auscultation was clear, cardiac examination revealed no murmurs, and abdominal examination showed active bowel sounds without tenderness. Mild erythema was noted at the chemoport insertion site, without associated swelling or discharge. Initial laboratory investigations were largely unremarkable, with a white blood cell count of 4690/mm^3^ and a C-reactive protein level of 0.3 mg/dL, both within normal limits, although a marked neutrophil predominance (94%) was observed.

On the third hospital day, blood cultures obtained simultaneously from peripheral blood and the chemoport yielded growth of *Leclercia adecarboxylata* and *Enterobacter cloacae* complex. Both organisms were identified using matrix-assisted laser desorption/ionization-time-of-flight mass spectrometry (MALDI-TOF MS; bioMérieux, Marcy-l’Étoile, France). Antimicrobial susceptibility testing was performed using the VITEK^®^2 system (bioMérieux) and interpreted according to Clinical and Laboratory Standards Institute (CLSI) breakpoints. The *L. adecarboxylata* isolate was susceptible to all tested antimicrobial agents, including trimethoprim–sulfamethoxazole, cefazolin, cefoxitin, ceftriaxone, ceftazidime, cefepime, cefoperazone-sulbactam, ampicillin-sulbactam, piperacillin-tazobactam, ciprofloxacin, ertapenem, imipenem, gentamicin, and amikacin. The *E. cloacae* complex isolate was susceptible to trimethoprim-sulfamethoxazole, ceftriaxone, ceftazidime, cefepime, cefoperazone-sulbactam, piperacillin-tazobactam, ciprofloxacin, ertapenem, imipenem, gentamicin, and amikacin, but resistant to cefazolin, cefoxitin, and ampicillin-sulbactam. The same organisms with identical antimicrobial susceptibility profiles were recovered from two sets of paired peripheral and catheter-drawn blood cultures.

The diagnosis of catheter-related bloodstream infection (CRBSI) was established based on accepted clinical and microbiological criteria, including isolation of identical organisms with matching susceptibility profiles from paired peripheral and catheter-drawn blood cultures in the absence of an alternative infectious focus. In addition, blood cultures drawn from the chemoport became positive two hours earlier than those obtained from peripheral blood, fulfilling the differential time to positivity (DTP) criterion for CRBSI. These findings strongly implicated the catheter as the source of infection. Intravenous ciprofloxacin (400 mg every 12 h) was initiated, and the chemoport was removed. Follow-up blood cultures obtained 72 h after catheter removal and initiation of antimicrobial therapy showed no bacterial growth. After completing 7 days of intravenous therapy, she was discharged with oral ciprofloxacin (500 mg every 12 h) for an additional 7 days.

## 3. Discussion

*Leclercia adecarboxylata* (formerly classified as the genus *Escherichia*) is a Gram-negative bacillus belonging to the Enterobacteriaceae family. In recent years, *L. adecarboxylata* has been increasingly recognized as an emerging opportunistic pathogen, particularly in immunocompromised individuals, as documented in multiple case reports (Table 1). The growing use of modern microbial identification technologies—most notably Matrix-Assisted Laser Desorption/Ionization-Time of Flight (MALDI-TOF)—has further contributed to its more frequent recognition by enabling accurate differentiation from similar *Escherichia* species [2]. Although infections caused by *L. adecarboxylata* remain uncommon, the organism is capable of inducing severe, potentially life-threatening disease. Continued research is needed to better understand its pathogenic mechanisms in humans.

*L. adecarboxylata* is a widely distributed microorganism that can be isolated from various environmental sources, including water and soil, and is also part of the normal intestinal microbiota in some animals [2]. *L. adecarboxylata* might also cause polymicrobial infection in immunocompromised patients. Table 1 summarizes previously reported cases of *L. adecarboxylata* polymicrobial bloodstream infections in the literature, among which our case represents the first reported instance of co-infection with *Enterobacter cloacae* complex (ECC). Similar to our case, most reported bloodstream infections have been associated with immunosuppression and catheter-related infections. Furthermore, several studies suggest that catheters may serve as important reservoirs for *L. adecarboxylata* bloodstream infections [4]. Similar observations have been reported in other CRBSI cases, where catheter removal was a decisive factor for clinical recovery.

**Table 1:** Clinical characteristics of patients with *Leclercia adecarboxylata* bloodstream co-infection.

Year/ Country	Age/ Gender	Underlying Diseases	Co-Infection Pathogen	Clinical Resolution	CRBSI	Reference
1995, Belgium	80, Female	Fourfold coronary bypass	*Escherichia hermannii*	N/A	No	[5]
1999, South Korea	69, Female	Leiomyosarcoma	*Escherichia hermannii*	N/A	Yes	[6]
2001, USA	11-month, Female	Acute lymphoblastic leukemia	*Staphylococcus aureus*	Clinical resolution with GEN and CZ	No	[7]
2009, Spain	72, Male	End-stage renal disease	*Pantoea agglomerans*	Clinical resolution with MEM	Yes	[8]
2017, Italy	50, Male	Human immunodeficiency virus and hepatitis C infection	*Enterococcus faecalis*	Clinical resolution with TZP	No	[9]
2021, USA	9-month, Male	Pompe disease	*Acinetobacter baumannii*	Clinical resolution	Yes	[10]
2025, Taiwan (This case)	54, Female	Breast cancer	*Enterobacter cloacae* complex	Clinical resolution with CIP	Yes	

Abbreviation: N/A, not available; CRBSI, catheter-related bloodstream infection; GEN, gentamicin; CZ, cefazolin; MEM, meropenem; TZP, piperacillin-tazobactam; CIP, ciprofloxacin.

A review of the literature (Table 2) shows that *L. adecarboxylata* CRBSI most frequently affects patients with end-stage renal disease or malignancy, with favorable outcomes once the catheter is removed and appropriate antibiotics are administered. Polymicrobial bloodstream infections involving *L. adecarboxylata* (Table 1) have occasionally included *Escherichia hermannii*, *Pantoea agglomerans*, *Enterococcus faecalis*, or *Acinetobacter baumannii*, but co-infection with ECC has not been previously documented. From a clinical perspective, *Enterobacter cloacae* complex is a well-recognized pathogen and a frequent cause of catheter-related bloodstream infections in hospitalized and immunocompromised patients. ECC is known for its ability to adhere to indwelling devices and form biofilms, as well as for its intrinsic and inducible antimicrobial resistance mechanisms, particularly chromosomal AmpC β-lactamase expression [3]. In the setting of co-infection, the presence of ECC may contribute to biofilm persistence and therapeutic complexity, further underscoring the importance of prompt catheter removal and appropriate antimicrobial selection.

Compared with previously reported cases of *Leclercia adecarboxylata* bloodstream co-infection, the clinical context and diagnostic certainty in earlier reports were often limited. In the study by de Baere et al. [5], *L. adecarboxylata* was isolated in mixed cultures with *Escherichia hermannii* from patients presenting with sepsis or chronic biliary infection; however, no definitive infectious focus or device-related source was identified, and the causal role of intravascular devices could not be established. Similarly, the case described by Lee et al. [6] reported *L. adecarboxylata* bacteremia associated with a Hickman catheter, yet the diagnosis of catheter-related bloodstream infection (CRBSI) was based primarily on culture results without comprehensive microbiological confirmation, such as paired peripheral and catheter-drawn blood cultures or differential time to positivity analysis. In these earlier reports, although polymicrobial isolation suggested a possible pathogenic role for *L. adecarboxylata*, the underlying mechanisms of co-infection remained speculative. The coexistence of these *Enterobacteriaceae* may reflect biofilm synergy on indwelling devices or transient translocation from colonized skin or mucosal sites, which merits further investigation, particularly in immunocompromised hosts or patients with long-term intravascular access.

Antimicrobial susceptibility testing in our case revealed full sensitivity of both isolates to most β-lactams, quinolones, and aminoglycosides. This finding is consistent with earlier reports showing that *L. adecarboxylata* remains broadly susceptible to commonly used antibiotics, although natural resistance to macrolides, rifampin, glycopeptides, and lincosamides has been described [20]. Nonetheless, sporadic reports of extended-spectrum β-lactamase (ESBL)- or New Delhi metallo-β-lactamase (NDM)-producing *L. adecarboxylata* indicate the potential for emerging resistance [21]. Continuous surveillance and molecular characterization of resistance determinants are therefore warranted, particularly in nosocomial settings.

Given the patient’s occupation as a housekeeper and frequent exposure to the hospital environment, environmental exposure, including contact with dust, contaminated water, or flushing solutions, could have contributed to colonization or contamination of the vascular access device. Although direct environmental cultures were not obtained, the clinical course and microbiological findings were consistent with a catheter-related source of infection. The successful management of this case with ciprofloxacin and prompt chemoport removal reinforces the importance of combining appropriate antimicrobial therapy with elimination of the infected device. As observed in prior reports, catheter removal remains the cornerstone of therapy for environmental Gram-negative CRBSI, as persistent biofilm colonization may lead to relapse despite in vitro susceptibility [4].

## 4. Conclusions

This case highlights the clinical relevance of *Leclercia adecarboxylata* and *Enterobacter cloacae* complex as potential pathogens capable of causing polymicrobial CRBSI in immunocompromised hosts. Clinicians should maintain a high index of suspicion for the rare organism when evaluating bloodstream infections in patients with indwelling catheters. Further genomic and environmental investigations are needed to elucidate its reservoirs, virulence factors, and interactions with other Enterobacterales species.

## Figures and Tables

**Table 2:** Clinical characteristics of patients with catheter-related bloodstream infections caused by *Leclercia adecarboxylata*.

Year/ Country	Age/ Gender	Underlying Diseases	Antibiotic Sensitivity	Antibiotic Resistance	Clinical Resolution	Catheter Removal	Reference
1999, South Korea	69, Female	Leiomyosarcoma	N/A	N/A	N/A	Yes	[6]
2009, Spain	81, Male	End-stage renal disease	N/A	FOS	Clinical resolution with CRO	No	[8]
2009, Spain	72, Male	End-stage renal disease	N/A	N/A	Clinical resolution with MEM	No	[8]
2011, USA	58, Male	End-stage renal disease	N/A	N/A	Clinical resolution	Yes	[11]
2012, USA	55, Male	Multiple trauma	AMP, CZ, FOX, CRO, CIP, GEN, TOB, SXT, AMK, FEP, SAM, IPM, TZP, ETP	N/A	Clinical resolution with CZ	Yes	[12]
2012, South Korea	47, Female	Breast cancer	AMC, TZP, FOX, CAZ, FEP, IPM, MEM, LVX, CIP, NOR, OFX, PEF, MXF, NA	AMP, PIP, ATM, CET, CZ, CCL, CTX, CFIX, CPZ, AMK, GEN, TOB, SXT	Clinical resolution with FEP	Yes	[13]
2013, Italy	81, Male	End-stage renal disease	Beta-lactams, third-generation cephalosporins, quinolones, TMP, aminoglycosides and carbapenems	N/A	Clinical resolution with GEN and AMC	No	[14]
2020, Saudi Arabia	50, Female	End-stage renal disease	CIP, GEN, AMK, MEM, SXT	AMC, AMP, CRO, TZP	Clinical resolution with GEN and MEM	N/A	[15]
2021, USA	9-month, Male	Pompe disease	N/A	N/A	Clinical resolution	Yes	[10]
2022, USA	34, Male	Soft tissue sarcoma	AMP, LVX, SXT	N/A	Clinical resolution with LVX	Yes	[16]
2023, Italy	38, Transgender woman	Gastric and duodenal diffuse large B-cell lymphoma	AMK, FEP, CTX, CAZ, CZA, C/T, CIP, CL, GEN, IPM, MEM, TZP, TOB	AMC, FOS, SXT	Clinical resolution with TZP	Yes	[17]
2023, India	50, Female	Breast cancer	FOX, carbapenems, AMK, GEN, CL, TGC	CIP, SXT	Clinical resolution with GEN and MEM	Yes	[18]
2023, India	68, Male	End-stage renal disease	FOX, FEP, AMC, CPZ/SUL, TZP, MEM, IPM, ETP, GEN, AMK, CL, PMB, TGC	AMP, first-generation cephalosporins, most second-generation cephalosporins, third-generation cephalosporins, CIP, OFX, LVX, SXT, ATM	Clinical resolution with GEN and MEM	Yes	[19]
2025, Taiwan (This case)	54, Female	Breast cancer	SXT, CZ, FOX, CRO, CAZ, FEP, CPZ/SUL, SAM, TZP, CIP, ETP, IPM, GEN, AMK	N/A	Clinical resolution with CIP	Yes	

Abbreviation: N/A, not available; FOS, fosfomycin; CRO, ceftriaxone; MEM, meropenem; AMP, ampicillin; CZ, cefazolin; FOX, cefoxitin; CIP, ciprofloxacin; GEN, gentamicin; TOB, tobramycin; SXT, trimethoprim-sulfamethoxazole; AMK, amikacin; FEP, cefepime; SAM, ampicillin-sulbactam; IPM, imipenem; TZP, piperacillin-tazobactam; ETP, ertapenem; AMC, amoxicillin-clavulanate; CAZ, ceftazidime; LVX, levofloxacin; NOR, norfloxacin; OFX, ofloxacin; PEF, pefloxacin; MXF, moxifloxacin; NA, nalidixic acid; PIP, piperacillin; ATM, aztreonam; CET, cephalothin; CCL, cefaclor; CTX, cefotaxime; CFIX, cefixime; CPZ, cefoperazone; TMP, trimethoprim; CZA, ceftazidime-avibactam; C/T, ceftolozane-tazobactam; CL, colistin; CPZ/SUL, cefoperazone-sulbactam.

## Data Availability

The original contributions presented in the study are included in the article, further inquiries can be directed to the corresponding author.

## References

[1] Gahlot R., Nigam C., Kumar V., Yadav G., Anupurba S. (2014). Catheter-related bloodstream infections. Int. J. Crit. Illn. Inj. Sci..

[2] Spiegelhauer M.R., Andersen P.F., Frandsen T.H., Nordestgaard R.L.M., Andersen L.P. (2019). *Leclercia adecarboxylata*: A case report and literature review of 74 cases demonstrating its pathogenicity in immunocompromised patients. Infect. Dis..

[3] Mezzatesta M.L., Gona F., Stefani S. (2012). *Enterobacter cloacae* complex: Clinical impact and emerging antibiotic resistance. Future Microbiol..

[4] Marco Lattur M.D., Payeras Cifre A., Bover Barceló I. (2010). Infection of reservoir-central venous catheter by *Leclercia adecarboxylata* in a patient with Ewing sarcoma. Med. Clin..

[5] De Baere T., Wauters G., Huylenbroeck A., Claeys G., Peleman R., Verschraegen G., Allemeersch D., Vaneechoutte M. (2001). Isolations of *Leclercia adecarboxylata* from a patient with a chronically inflamed gallbladder and from a patient with sepsis without focus. J. Clin. Microbiol..

[6] Lee N.Y., Ki C.S., Kang W.K., Peck K.R., Kim S., Song J.H. (1999). Hickman catheter-associated bacteremia by *Leclercia adecarboxylata* and *Escherichia hermannii*: A case report. Korean J. Infect. Dis..

[7] Longhurst C.A., West D.C. (2001). Isolation of *Leclercia adecarboxylata* from an infant with acute lymphoblastic leukemia. Clin. Infect. Dis..

[8] Fernández-Ruiz M., López-Medrano F., García-Sánchez L., García-Reyne A., de Solo T.O., Sanz-Sanz F., Aguado J.M. (2009). Successful management of tunneled hemodialysis catheter-related bacteremia by *Leclercia adecarboxylata* without catheter removal: Report of two cases. Int. J. Infect. Dis..

[9] Saccani B., Izzo I., Sasari S., Magro P., Ravizzola G., Spinetti A., Castelli F. (2017). *Leclercia adecarboxylata* isolation from blood cultures: An emerging pathogen in immunocompromised hosts?. Infect. Dis. Trop. Med..

[10] Saadoon R., Akinsanmi D., Xu S., Alharash H., Hammerschlag M. (2021). *Leclercia adecarboxylata*: A Rare Cause of CRBSI in a Child with Pompe Disease. Crit. Care Med..

[11] Marina V.P., Abidi S., Malhotra D. (2011). *Leclercia adecarboxylata*, an unusual hemodialysis catheter-related infection. Int. Urol. Nephrol..

[12] Forrester J.D., Adams J., Sawyer R.G. (2012). *Leclercia adecarboxylata* bacteremia in a trauma patient: Case report and review of the literature. Surg. Infect..

[13] Shin G.W., You M.J., Lee H.S., Lee C.S. (2012). Catheter-related bacteremia caused by multidrug-resistant *Leclercia adecarboxylata* in a patient with breast cancer. J. Clin. Microbiol..

[14] De Mauri A., Chiarinotti D., Andreoni S., Molinari G.L., Conti N., De Leo M. (2013). *Leclercia adecarboxylata* and catheter-related bacteraemia: Review of the literature and outcome with regard to catheters and patients. J. Med. Microbiol..

[15] Alosaimi R.S., Muhmmed Kaaki M. (2020). Catheter-Related ESBL-Producing *Leclercia adecarboxylata* Septicemia in Hemodialysis Patient: An Emerging Pathogen?. Case Rep. Infect. Dis..

[16] Harper H., Logan J., Kubat R., Jones M. (2022). *Leclercia adecarboxylata* catheter-related bacteraemia in an immunocompromised patient. BMJ Case Rep..

[17] Colangelo C., Tiecco G., Di Gregorio M., Capone S., Allegri R.L., De Francesco M., Caccuri F., Caruso A., Castelli F., Focà E. (2023). A Rare Case of Multidrug-resistant *Leclercia adecarboxylata* Catheter-related Bloodstream Infection and an Updated Brief Literature Review. Mediterr. J. Hematol. Infect. Dis..

[18] Sardana V., Verma S.R. (2023). *Leclercia adecarboxylata*: An Emerging Multidrug Resistant Pathogen Causing Catheter Related Bacteremia with Review of Literature. J. Dent. Med. Sci..

[19] Sardana V., Verma S.R. (2023). Catheter-related bloodstream infection caused by an extended-spectrum beta-lactamase (ESBL) producing *Leclercia adecarboxylata* in a hemodialysis patient. Paripex Indian J. Res..

[20] Stock I., Burak S., Wiedemann B. (2004). Natural antimicrobial susceptibility patterns and biochemical profiles of *Leclercia adecarboxylata* strains. Clin. Microbiol. Infect..

[21] Riazzo C., López-Cerero L., Rojo-Martín M.D., Hoyos-Mallecot Y., Fernández-Cuenca F., Martín-Ruíz J.L., Pascual-Hernández Á., Naas T., Navarro-Marí J.M. (2017). First report of NDM-1-producing clinical isolate of *Leclercia adecarboxylata* in Spain. Diagn. Microbiol. Infect. Dis..

